# A Micro Dynamically Tuned Gyroscope with Adjustable Static Capacitance

**DOI:** 10.3390/s130202176

**Published:** 2013-02-06

**Authors:** Dunzhu Xia, Cheng Yu, Lun Kong

**Affiliations:** Key Laboratory of Micro-inertial Instrument and Advanced Navigation Technology, Ministry of Education, Southeast University, Nanjing 210096, China; E-Mails: 101010203@seu.edu.cn (C.Y.); lunkong2013@163.com (L.K.)

**Keywords:** micro dynamically tuned gyroscope (MDTG), rotor wafer, adjustable capacitance, digitalized correction and decoupling

## Abstract

This paper presents a novel micro dynamically tuned gyroscope (MDTG) with adjustable static capacitance. First, the principle of MDTG is theoretically analyzed. Next, some simulations under the optimized structure parameters are given as a reference for the mask design of the rotor wafer and electrode plates. As two key components, the process flows of the rotor wafer and electrode plates are described in detail. All the scanning electron microscopy (SEM) photos show that the fabrication process is effective and optimized. Then, an assembly model is designed for the static capacitance adjustable MDTG, whose static capacitance can be changed by rotating the lower electrode plate support and substituting gasket rings of different thicknesses. Thus, the scale factor is easily changeable. Afterwards, the digitalized closed-loop measurement circuit is simulated. The discrete correction and decoupling modules are designed to make the closed-loop stable and cross-coupling effect small. The dual axis closed-loop system bandwidths can reach more than 60 Hz and the dual axis scale factors are completely symmetrical. All the simulation results demonstrate the proposed fabrication of the MDTG can meet the application requirements. Finally, the paper presents the test results of static and dynamic capacitance values which are consistent with the simulation values.

## 1. Introduction

As a traditional high precision gyroscope (the bias stability is usually around 0.001–1 °/h), the dynamically tuned gyroscope (DTG) has been widely applied in the fields of inertial navigation and accurate positioning. The DTG signals contain noise and random drift due to the influence of the rebalance loop, driving motor and the structural thermal deformation, *etc*. In [1–3], the wavelet transform method was used in filters to reduce the random drift and noise. The empirical mode decomposition (EMD) method is another way for DTG signal denoising [4,5]. In addition to the disadvantages of random drift and noise, the traditional DTGs are voluminous, heavy, expensive and their operation is complicated. As silicon Micro-electromechanical Systems (MEMS) have developed, micro gyroscopes have recently been developed in the commercial field as a kind of miniaturized angular rate sensor for many applications like rollover detection, inertial navigation, and electronic stability programs [6]. Compared with traditional gyroscopes, MEMS gyroscopes are small in volume, lightweight, low in cost and easy to mass produce. By adopting micromachining technology, the traditional DTG has evolved into a novel micro gyroscope which can simultaneously meet the requirements of miniaturization from MEMS gyroscope and high precision from DTG [7].

The research on the MDTG was first proposed by Jenkins *et al*. [8] in 2003. In [9], the MDTG was briefly described and fabricated. In order to realize the dynamical tuning, a bias voltage is applied to the gyroscope which is different from the traditional DTG. Another difference is the detection method where a capacitance signal detection is applied to the MDTG. The static capacitance of the previous MDTG is constant in the assembly model and there are no details in regard to its key fabrication technology and its assembly model. In order to further improve the performance of the gyro, high precision fabrication should be focused on. In this work, we will discuss in detail the MDTG fabrication including the electrode plate, rotor wafer and its assembly model. The new assembly has advantages of conveniently changeable static capacitance over the previous design.

Regardless of the difference between the DTG and MDTG, normally we can adopt the circuitry and signal processing methods in the tradional DTG as a key reference of the MDTG concerning the rebalance loop. In recent years, the rebalance loop has been designed for the DTG using analog or digital circuits, where a lot of control and signal processing approaches are investigated to improve the performance of the DTG [10,11]. As known, a new interface circuit should be designed to realize the capacitance signal extraction and electric stiffness tuning. The rebalance loop of the MDTG has been designed using a pure analog circuit in [9], whereas with analog circuits it is difficult to meet the requirements in terms of complexity, flexibility, and intelligent compensation [12]. In order to further improve the performance of the MDTG, a digital circuit should be used in the external signal processing of the gyroscope. In this paper, a digital rebalance loop is designed for a static capacitance adjustable MDTG to solve the problems mentioned above.

### 2. Principle of the MDTG

**Figure 1.**
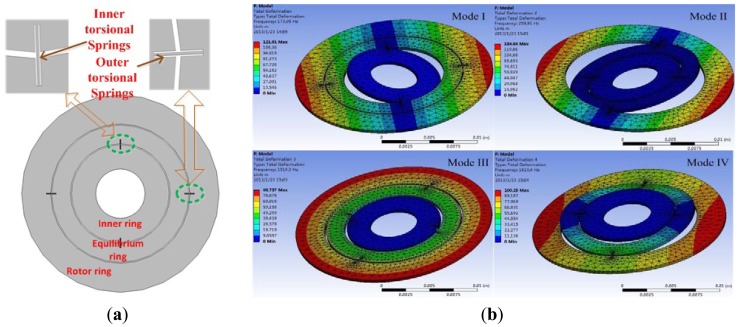
(**a**) The rotor wafer. (**b**) The modal simulation.

The rotor wafer of the MDTG in Figure 1(a) is driven by a miniature motor to rotate rapidly. When an angular rate along the x-axis or y-axis occurs, the rotor wafer deflects, causing a change of capacitance values between the electrode plates and the rotor wafer. Thus, the input angular rate can be measured according to the change of capacitance values. To maintain the balance position of the rotor disc, an electrostatic feedback moment should be applied on the rotor wafer to rectify the rotor wafer deflection. A bias voltage is applied on the rotor wafer to achieve dynamical tuning because of the lack of the equilibrium ring negative stiffness. According to [13], neglecting some secondary factors, the MDTG kinematic equations can be expressed as:

(1) $$\{\begin{array}{l} J\ddot{\beta }+\delta \dot{\beta }+(\Delta K-K_{C})\beta +H\dot{\alpha }+\lambda \alpha =M_{x}-J{\ddot{\varphi }}_{x}-H{\dot{\varphi }}_{y}\\ J\ddot{\alpha }+\delta \dot{\alpha }+(\Delta K-K_{C})\alpha -H\dot{\beta }-\lambda \beta =M_{y}-J{\ddot{\varphi }}_{y}-H{\dot{\varphi }}_{x} \end{array}$$

where *J* is the moment of inertia of the gyroscope. H is the moment of momentum of the MDTG, Δ*K* = *K_p_* − *K_n_* is the remaining stiffness. *K_p_* is the positive torsional rigidity coefficient of the torsional springs. *K_n_* is the negative stiffness coefficient of the equilibrium ring. *K_C_* is the electric stiffness coefficient generated by the bias voltage. α and β are the angular displacements of the rotation axes of the rotor wafer around the x-axis and y-axis, respectively. *M_x_* and *M_y_* are the feedback moments of the rotor wafer along x-axis and y-axis, respectively. φ̇_x_ and φ̇_y_ are respectively the input rotational rate along y-axis and x-axis. λ, δ and *D* are the damping coefficients.

## 3. Simulation of the MDTG

### 3.1. Modal Simulation

The MDTG structure adopted here is shown in Figure 1. The inner ring is rigidly attached to the micro motor driving shaft. The equilibrium ring and rotor ring are free to rotate around the inner and outer beams. When the rotor rotates at a high speed, up to more than 10,000 rpm, the equilibrium ring and rotor ring will get a sufficient and stable moment of inertia. In an ideal situation, the so called dynamically tuned condition, *i.e*., theoretically the negative stiffness of equilibrium ring and tuning electric current can cancel out the positive stiffness of inner and outer torsion bars. The rotor ring will keep the characteristic of the gyroscope's stable spin axis. As the outer angular rate occurs along the x-axis and y-axis, the precession will respond proportionally and simultaneously.

During this process, the equilibrium ring always do a rocking motion at twice the frequency of the rotation rate. To make the rotor ring work in a stable and limited area, the closed-loop force rebalance mechanism is adopted to offset its rotation in the centre position. Here the negative electrical stiffness and the feedback torque will simultaneously be generated from a higher servo voltage originated by the correction and decoupling operation of two angle signals of the rotor rings around x and y axes. To make sure that all the modes are effectively away from the second and third harmonic frequencies of the motor rotation rate, we need to select optimized geometry parameters to get more proper modes.

The meshing method of the torsional spring and other parts are adopted by 1 μm fine and 5 μm normal tetrahedron meshing elements, respectively. The center inner cylinder of the inner ring is fixed as a boundary condition.Through modal simulation in Figure 1(b), the first six modes are attained, including mode I rotation around the inner torsion bar at 173.95 Hz, mode II rotation around the outer torsion bar at 259.91 Hz, mode III rotation in the plane at 1,514.3 Hz, mode IV vibration up and down at 1,823.4 Hz and other harmonic vibrations along the right and left sides of the inner torsion bar at 2601.2 Hz, vibration along the right and left sides of inner torsion bar at 4,744.4 Hz. Considering the motor rotation rate of 10,000 rpm, we find that all these modes are not overlapped by its harmonic frequencies.

### 3.2. Mechanical Simulation

The MDTG mechanical simulation is necessary for optimizing the structure parameters in Table 1. The deformation of the MDTG must be controlled within less than 1 μm while the deformation of the torsional spring must be tiny enough in any cases. The field equations used in the mechanical simulation can be written as:

(2) $$\{\begin{array}{l} -\nabla \cdot \sigma =F_{V}\\ \varepsilon =f(\sigma )\\ \varepsilon =\frac{1}{2}[{\left(\nabla U\right)}^{T}+\nabla U] \end{array}$$

where ∇ represents $\left[\frac{\partial }{\partial x}\;\frac{\partial }{\partial y}\;\frac{\partial }{\partial z}\right]$, stress matrix $\sigma =\left[\begin{array}{ccc} \sigma_{\text{x}} & \tau_{\text{yx}} & \tau_{\text{zx}}\\ \tau_{\text{xy}} & \sigma_{\text{y}} & \tau_{\text{zy}}\\ \tau_{\text{xz}} & \tau_{\text{yz}} & \sigma_{\text{z}} \end{array}\right]$, strain matrix $\varepsilon =\left[\begin{array}{ccc} \varepsilon_{\text{x}} & \frac{\gamma_{\text{xy}}}{2} & \frac{\gamma_{\text{xz}}}{2}\\ \frac{\gamma_{\text{xy}}}{2} & \varepsilon_{\text{y}} & \frac{\gamma_{\text{yz}}}{2}\\ \frac{\gamma_{\text{xz}}}{2} & \frac{\gamma_{\text{yz}}}{2} & \varepsilon_{\text{z}} \end{array}\right]$, displacement matrix $\text{U}=\left[\begin{array}{c} \text{u}\\ \text{v}\\ \text{w} \end{array}\right]$, body force matrix ${\text{F}}_{\text{V}}=\left[\begin{array}{c} {\text{f}}_{\text{x}}\\ {\text{f}}_{\text{y}}\\ {\text{f}}_{\text{z}} \end{array}\right]$. ***ε*** = *f*(***σ***) is the physical equation between stress and strain which is related to Young's modulus and Poisson's ratio. When the initial stress, strain, displacement or body force matrixes are given, the displacement of the rotor wafer structure can be obtained.

**Figure 2.**
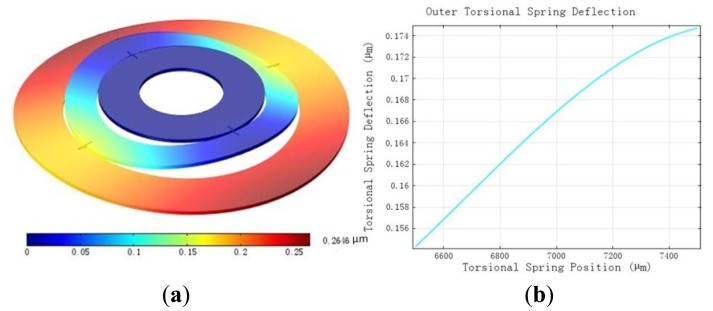
The deflection under gravity. (**a**) The rotor wafer deflection. (**b**) The torsional spring deflection.

According to the field Equation (2), the MDTG deflection under gravity can be seen in Figure 2(a). The inner ring is fastened to the driving shaft of the miniature motor. The whole rotor wafer has the downward body force under gravity. Using tetrahedral mesh, the maximum deflection is 0.2646 μm, and appears at the edge of the rotor ring. Figure 2(b) shows the deflection of the outer torsional spring. The deflection of the inner torsional spring is smaller, as seen in Figure 2(a). The maximum deflection of the torsional spring is 0.176 μm.

**Figure 3.**
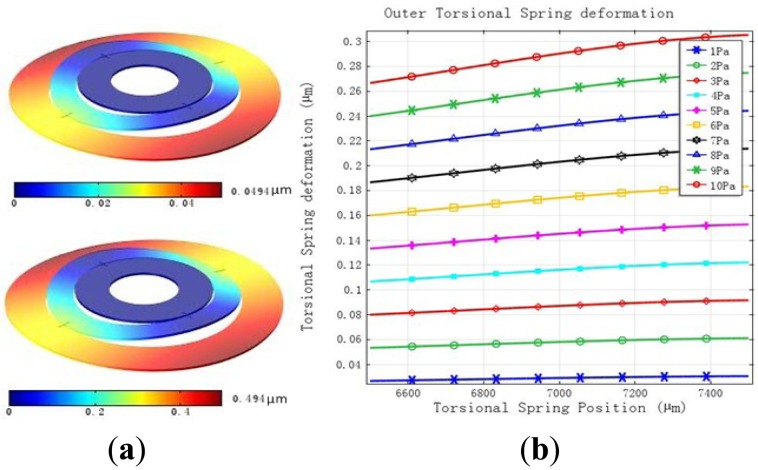
The deformation under a load. (**a**) The rotor wafer deformation. (**b**) The torsional spring deformation.

The rotor ring of MDTG should have some load capacity especially used in reality. The deformation when a pressure of 1 Pa to 10 Pa is applied on the upside of the rotor ring while the inner ring is constant can be seen in Figure 3. The maximum deformation changes from 0.0494 μm of 1 Pa to 0.494 μm of 10 Pa which can meet the requirements of less than 1 μm in Figure 3(a). The deformation of the outer torsional springs is larger than the inner ones. As the load continues to increases the deformation of the torsional spring becomes large obviously in Figure 3(b).

**Figure 4.**
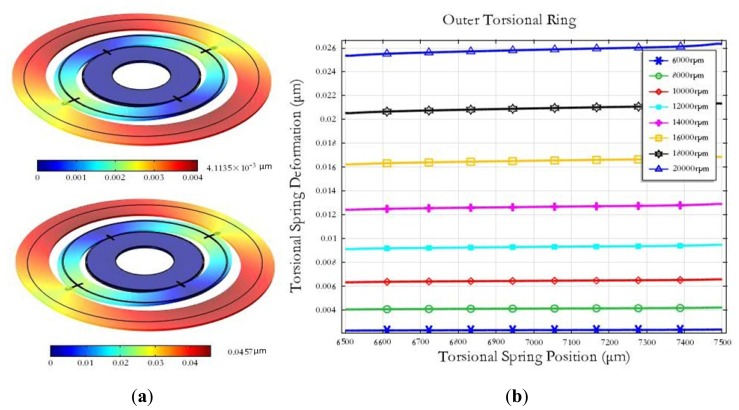
The deformation under a rotation speed. (**a**) The rotor wafer deformation. (**b**) The torsional spring deformation.

The miniature motor used in the MDTG can spin from 6,000 rpm up to 20,000 rpm. When the MDTG rotates around the z-axis quickly, the rotor wafer will be subject to the force in the xy planes caused by the centrifugal force. As the rotation speed ranges from 6,000 rpm to 20,000 rpm, the maximum deformation can reach from 0.0041 μm at 6,000 rpm to 0.0457 μm at 20,000 rpm in Figure 4(a). The deformation of the outer torsional spring will rise rapidly as the rotation speed increases in Figure 4(b). The simulation results show that the rotation deformation is smaller than the other mechanical simulations and the rotation speed can be set higher.

### 3.3. Static Capacitance Simulation

**Figure 5.**
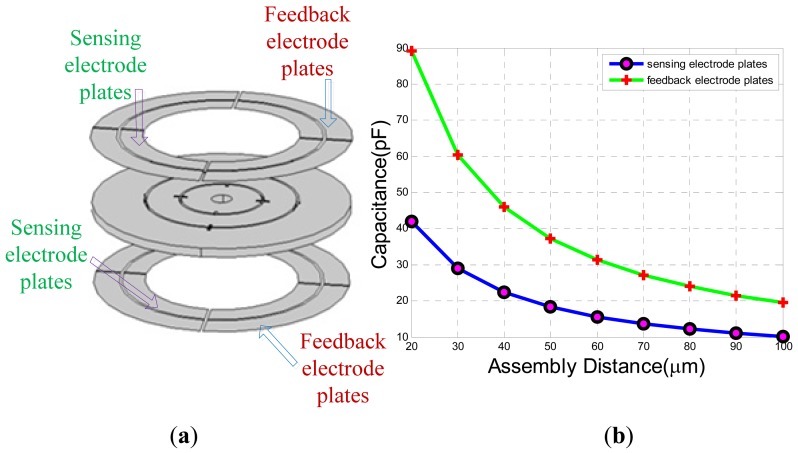
The simulation of static capacitance values. (**a**) The rotor wafer and the electrode plates. (**b**) The diagram of static capacitance simulation.

The assembly distance between the electrode plates and the MDTG is very vital to the capacitance signal extraction shown in Figure 5(a). There are two electrode plates for differential detection. The outer rings of the two electrode plates are feedback electrode plates and the inner rings are sensing electrode plates. When the MDTG has a tiny declination, the capacitances between the sensing electrode plates and the rotor ring will change. The carrier signal and feedback voltage can be applied on the feedback electrode plates to form a rebalance closed-loop control. The capacitance value will decrease naturally when the assembly distance increases. The specific capacitance values between the electrode plates and the rotor ring are shown in Figure 5 when the assembly distance changes from 20 μm to 100 μm. Thus, the capacitance values of the sensing electrode plates range from 40 pF to 10 pF. Meanwhile, the capacitance values of the feedback electrode plates range from 90 pF down to 20 pF. Considering the rotor wafer rotation deflection angle working at the open-loop and the capacitance values, here the assembly distance is chosen from 40 μm to 60 μm.

## 4. Fabrication of MDTG

### 4.1. Fabrication of the Rotor Wafer

**Figure 6.**
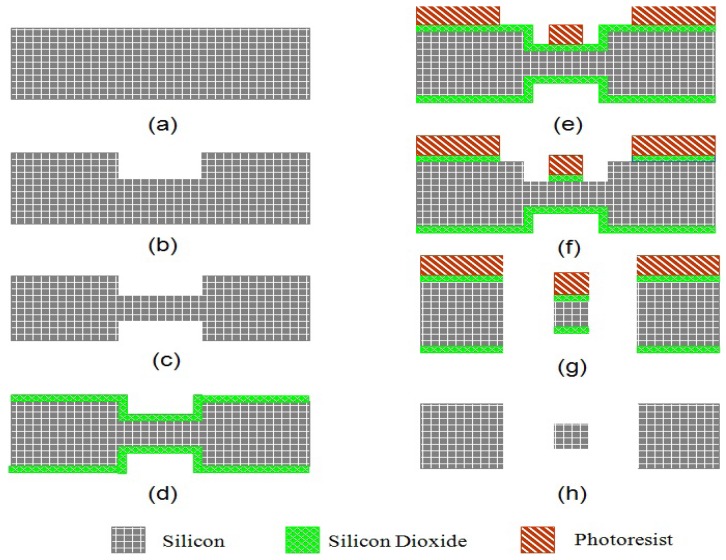
Process flow for Rotor.

The fabrication process starts with a double side polished boron doped low resistivity silicon wafer shown as Figure 6(a). The thickness of torsion beam is defined by a shallow etch of bulk silicon from both sides of the wafer. Adhesion promoter layer hexamethyldisilazane (HMDS) is firstly spun at 3,000 rpm for 35 seconds and then baked at 115 °C for 30 seconds to improve the adhesion strength between photoresist and silicon. Next Shipley 1827 positive photoresist is spun at 3,000 rpm for 35 seconds to achieve the thickness of 2.7 μm. It is then soft baked at 115 °C for 3 minutes. UV exposure at 405 nm for 700 mJ/cm^2^ is conducted to pattern the opening for selective etching bulk silicon. MF-319 is used to develop the UV exposed photoresist. The shallow trench is etched in high aspect ratio Bosch ICP etching to ensure straight sidewalls as seen in Figure 6(b). A trench depth of 25 μm is achieved by 25 cycles of etching.

The same recipe is used for the photoresist on the back side of the wafer in Figure 6(c). By performing the front side to the back side alignment, the thickness of the torsional beam is defined. After the shallow trench is etched from both the front side and back side, silicon dioxide is thermally grown at 1,100 °C to achieve the thickness of 4 μm. They serve as the masking layer for final through silicon trench etching in Figure 6(d). In order to have good photoresist coverage above the shallow trench in the next lithography step, AZ 4620 positive photoresist is used to obtain a very thick photoresist layer in Figure 6(e). Thickness of 20 μm is achieved by spinning AZ 4620 at 800 rpm and soft baking at 115 °C for 6 minutes. UV exposure of 2,000 mJ/cm^2^ light intensity at 405 nm can pattern the photoresist in Figure 6(f). Photoresist AZ 400K that mixed with DI water by the volume ratio of 1:3 is used to develop the pattern.

After AZ 4620 photoresist is developed, the thermally grown silicon dioxide masking layer is etched in C_4_F_8_ plasma. The remaining photoresist and silicon dioxide are the masking layers for the through silicon wafer etch. Then the bulk silicon is etched by Bosch process to ensure straight sidewalls in Figure 6(g). The last step of the fabrication of rotor is removing the remaining photoresist in acetone and the thermal oxide in hydrofluoric acid (HF), respectively, as seen in Figure 6(h).

**Figure 7.**
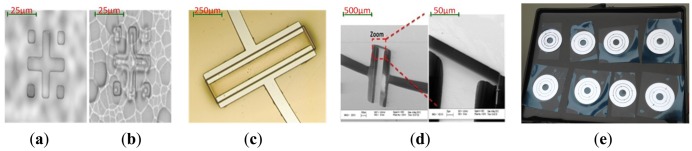
Views of fabrication. (**a**) Backside alignment under IR microscope when focus is on back side without IR. (**b**) Backside alignment with IR. (**c**) AZ 4620 pattern after developing. (**d**) SEM of the torsion rod. (**e**) The photo of the rotor wafers.

During the above process, some key characteristics are recorded by microscope diagrams. Figure 7(a) shows the alignment mark of the backside under IR (infrared) microscope, which is transparent to silicon. Photoresist pattern at front side is shown while the focus is on the front side of the wafer with IR turned off. In Figure 7(b), the focus can be shown on the back side of the wafer while IR is turned on. Both the front side and back side patterns can be observed and a very precise alignment (misalignment<3 μm) is valid. Figure 7(c) demonstrates the developed photoresist on the wafer with a shallow trench. As can be seen, there is no bubbles result from the topography of the surface and a very good coverage is also shown. Figure 7(d) shows the SEM image of the torsion beam with 25 μm recess on the silicon. Figure 7(e) shows the photo of the rotor wafers.

### 4.2. Fabrication of the Electrode Plates

**Figure 8.**
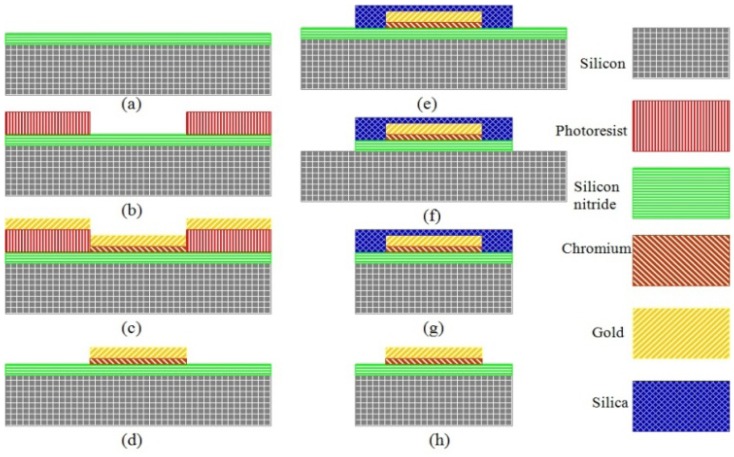
Process flow for electrode plate.

Figure 8 shows us the process flow of the electrode plates. First in Figure 8(a), a 10 um layer silicon nitride is deposited by LPCVD to be insulated from the base silicon. In Figure 8(b), a concave is formed by the exposure and development of the photoresist. In Figure 8(c), a 10 nm thin Cr layer as an adension is successfully sputtered and then removed by polishing at two sides. Next a 200 nm thick gold electrode layer is sputtered on the top. In Figure 8(d), the lift-off technology is used only to leave the middle part of metal layer and realize self-alignment of the overlayers. Then, in Figure 8(e), a 10 um thick layer of silicon dioxide is deposited by PECVD as an important mask for ICP etching of silicon nitride in Figure 8(f) and silicon in Figure 8(g). Last acetone and HF will be used, respectively, to remove the silicon dioxide, (Figure 8(h)).

**Figure 9.**
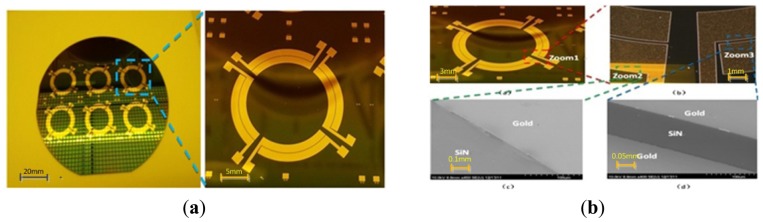
(**a**) SEM photos of electrode plate wafer. (**b**) SEM photos of detailed electrode plate parts.

Figure 9(a,b) demonstrate the final fabrication effects. One wafer can contain six pieces of electrode plates. The boundaries between silicon nitride and gold are very distinct. Besides, the electrical conductivity is qualified by the multimeter measurement.

### 4.3. Fabrication of the Assembly Model

**Figure 10.**
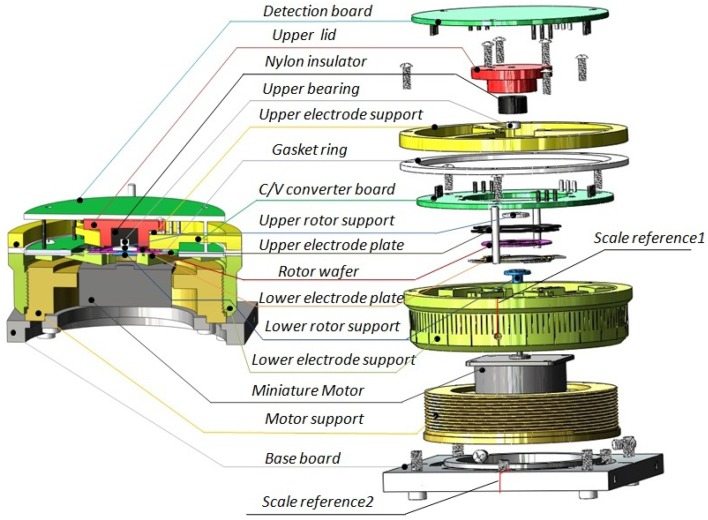
The assembly model of MDTG.

A novel assembly model shown in Figure 10 is developed and fabricated to achieve the static adjustable capacitance. The assembly consists of the base board, motor support, miniature motor, lower electrode support, lower rotor support, lower electrode plates, rotor wafer, upper electrode plate, upper lower support C/V converter board, gasket ring, upper electrode support, upper bearing, nylon insulator, upper lid, detection board and other assembly parts such as bolts and screws.

The base board is connected to the motor support through a chute. Thus, the base board could rotate freely to ensure that the coordinates of the base board are in accordance with the coordinates of the electrode plates for the purpose of convenient adjustment. The lower electrode support and the motor support are assembled together through the screw thread so that the gap between the rotor wafer and the lower electrode plate can be adjusted by screwing. There are total 120 even scales on the lower electrode support. When the scale reference 1 and the scale reference 2 are aligned, the assembly gap is zero. Then, the adjustable gap increases 4.167 μm by rotating a scale. The capacitance values are changeable with the gap shown in Figure 5(a). The gap between the upper electrode plate and rotor wafer can be adjusted via the gasket ring with the different thickness. The gap can be written as:

(3) $${\text{H}}_{2}={\text{D}}_{1}‐{\text{H}}_{1}‐{\text{D}}_{2}$$

where D_1_, D_2_ are the thicknesses of gasket ring and C/V converter board, respectively. H_1_, H_2_ are the gaps of the lower and upper electrode plates, respectively. The rotor wafer is fixed by the lower rotor support and upper rotor support. The signals such as carrier signal and bias voltage can be applied on the rotor wafer through upper bearing even though the rotor wafer rotates at a high speed. Besides, the C/V converter board can be embedded in the assembly and all the interface leads are symmetrically distributed by two layers beneficial for soldering, which can greatly eliminate the parasitic effect to improve the anti-interference capability between the signals. The adjustable capacitance assembly obviously has the advantage of adjusting the scale factor easily, avoiding pull in effect and so on.

## 5. Simulation of the Digitalized Closed-Loop Detection Circuit

### 5.1. Design of Digital System

As seen from Equation (1), the MDTG is actually a two-degree of freedom gyroscope and there are two loops called x-axis and y-axis loops in the system. To realize the dynamical tuning, the bias voltage in this system should be added to the feedback electrode plates to keep Δ*K* – *K_C_* zero. *K*_C_ can be written as:

(4) $$K_{\text{C}}=\frac{\varepsilon \gamma ({\text{R}}_{\text{r}}^{4}-{\text{r}}_{\text{F}}^{4}){\text{V}}_{\text{F}}^{2}}{{\text{d}}^{3}}$$

where ε is the dielectric constant of air, γ is the radian of single feedback electrode plate, R_r_ is the outer radius of the rotor ring, r_F_ is the inner radius of the feedback electrode plate, V_F_ is the bias voltage and d is the assembly gap between the electrode plates and the rotor wafer. Therefore, the bias voltage is:

(5) $${\text{V}}_{\text{F}}=\sqrt{\frac{{\text{d}}^{3}({\text{K}}_{\text{p}}-{\text{K}}_{\text{n}})}{\varepsilon \gamma ({\text{R}}_{\text{r}}^{4}-{\text{r}}_{\text{F}}^{4})}}$$

**Figure 11.**
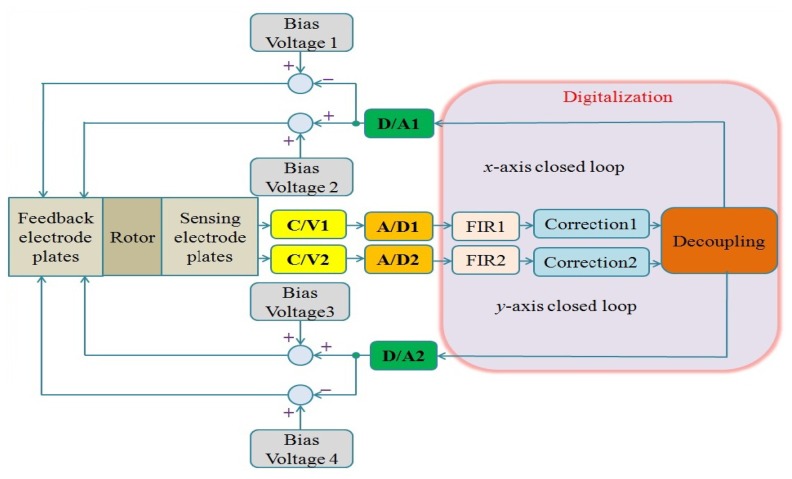
The digital MDTG circuitry system.

The digital circuit of MDTG can be seen in Figure 11, where FIR, correction and decoupling modules are digitally designed based on FPGA. The C/V converters with the diode ring demodulation can simplify the MDTG circuitry system without degradation of the overall performance.

### 5.2. C/V Converter with Demodulation

**Figure 12.**
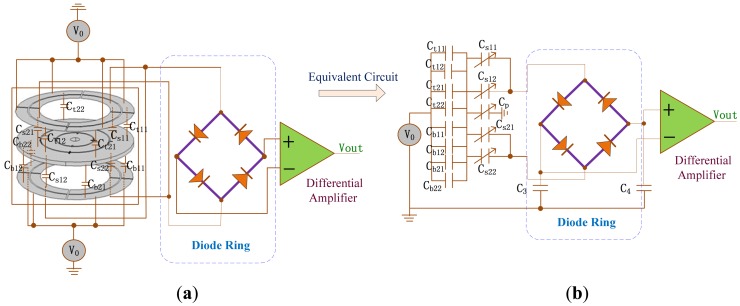
The interface circuit of MDTG. (**a**) Interface schematic diagram. (**b**) Equivalent circuit diagram.

The sensing electrode plates and feedback electrode plates are upper and lower electrode plates in Figure 5(a). C/V circuit with diode ring demodulation and its interface equivalent circuit can be seen in Figure 12(a). *C_t11_*, *C_t12_*, *C_t21_* and *C_t22_* are the capacitances between the upper feedback electrode plates and the rotor wafer while *C_b11_*, *C_b12_C_b21_* and *C_b22_* are the capacitances between the lower feedback electrode plate and the rotor wafer. *V_0_* is square wave carrier signal. The sensing capacitances *C_s11_*, *C_s12_*, *C_s21_* and *C_s22_* are formed between the sensing electrode plates and the rotor wafer. According to equivalent circuit in Figure 12(b), the output can be expressed as:

(6) $$\text{Vout}=-2K_{a}(V_{0}–V_{D})\frac{C_{0}}{{\left(C_{0}+C_{s11}+C_{s12}\right)}^{2}}\frac{C_{t11}+C_{t12}+C_{t21}+C_{t22}+C_{b11}+C_{b12}+C_{b21}+C_{b22}}{2\frac{C_{0}(C_{s11}+C_{s12})}{C_{0}+C_{s11}+C_{s12}}+C_{p}+C_{t11}+C_{t12}+C_{t21}+C_{t22}+C_{b11}+C_{b12}+C_{b21}+C_{b22}}\Delta C$$

where *C_3_=C_4_=C_0_* and *C_s11_+C_s12_=C_s21_+C_s22_*. *V_0_* is a high frequency carrier signal with frequency of 4 MHz and amplitude of 2.5 V while *V_D_* is diode drop voltage. Δ*C* is the total capacitance change of *C_s11_* and *C_s12_C_p_* is the parasitic capacitance. *K_a_* is the amplification factor of the differential amplifier. The interface circuit can greatly save the resources of FPGA without demodulation algorithm and eliminate the common mode noise interference.

### 5.3. Correction and Decoulping

When Δ*K* − *K_C_* = 0 under a certain bias voltage, the gyroscope will work at the dynamical tuning status and Equation (1) can be simplified without considering the damping torques as:

(7) $$\left[\begin{array}{c} \beta (s)\\ \alpha (s) \end{array}\right]=G(s)\left[\begin{array}{c} M_{x}(s)\\ M_{y}(s) \end{array}\right]-\left[\begin{array}{c} \varphi_{x}(s)\\ \varphi_{y}(s) \end{array}\right]$$

(8) $$G(s)=\left[\begin{array}{cc} \frac{1/J}{s^{2}+{\left(H/J\right)}^{2}} & \frac{-H/J^{2}}{s[s^{2}+{\left(H/J\right)}^{2}]}\\ \frac{H/J^{2}}{s[s^{2}+{\left(H/J\right)}^{2}]} & \frac{1/J}{s^{2}+{\left(H/J\right)}^{2}} \end{array}\right]$$

The decoupled transfer function matrix can be set as type II:

(9) $$G\prime (s)=\left[\begin{array}{cc} G\prime_{11}(s) & 0\\ 0 & G\prime_{22}(s) \end{array}\right]=\left[\begin{array}{cc} \frac{1}{Hs^{2}} & 0\\ 0 & \frac{1}{Hs^{2}} \end{array}\right]$$

With a zero-order holder, a new Z transformation equation can be written as:

(10) $$\left[\begin{array}{cc} G\prime_{11}(z) & 0\\ 0 & G\prime_{22}(z) \end{array}\right]=\left[\begin{array}{cc} \frac{T^{2}(z+1)}{2H{\left(z-1\right)}^{2}} & 0\\ 0 & \frac{T^{2}(z+1)}{2H{\left(z-1\right)}^{2}} \end{array}\right]$$

Considering the zero-order holder, we can obtain *G*(*z*) from *G*(*s*) through Z-transformation:

(11) $$G(z)=Z[\frac{1-e^{-Ts}}{s}G(s)]=\left[\begin{array}{cc} G_{11}(z) & G_{12}(z)\\ G_{21}(z) & G_{22}(z) \end{array}\right]$$

Let *Y*(*z*) represent the output of the decoupling part, then:

(12) $$Y(z)=\left[\begin{array}{c} \beta (z)\\ \alpha (z) \end{array}\right]=\left[\begin{array}{cc} D_{11}(z) & D_{12}(z)\\ D_{21}(z) & D_{22}(z) \end{array}\right]\left[\begin{array}{cc} G_{11}(z) & G_{12}(z)\\ G_{21}(z) & G_{22}(z) \end{array}\right]\left[\begin{array}{c} U_{1}(z)\\ U_{2}(z) \end{array}\right]$$

where $D(z)=\left[\begin{array}{cc} D_{11}(z) & D_{12}(z)\\ D_{21}(z) & D_{22}(z) \end{array}\right]$ is the desired decoupling matrix. The decoupled system has a characteristic of diagonal matrix, thus we can assume that:

(13) $$\left[\begin{array}{cc} D_{11}(z) & D_{12}(z)\\ D_{21}(z) & D_{22}(z) \end{array}\right]\left[\begin{array}{cc} G_{11}(z) & G_{12}(z)\\ G_{21}(z) & G_{22}(z) \end{array}\right]=\left[\begin{array}{cc} G_{11}^{\prime }(z) & 0\\ 0 & G_{22}^{\prime }(z) \end{array}\right]$$

Solving the equation above, we can obtain the digital decoupling matrix *D*(*z*):

(14) $$D(z)=\left[\begin{array}{cc} D_{11}(z) & D_{12}(z)\\ D_{21}(z) & D_{22}(z) \end{array}\right]={\left[\begin{array}{cc} G_{11}(z) & G_{12}(z)\\ G_{21}(z) & G_{22}(z) \end{array}\right]}^{-1}\left[\begin{array}{cc} G_{11}^{\prime }(z) & 0\\ 0 & G_{22}^{\prime }(z) \end{array}\right]$$

**Figure 13.**
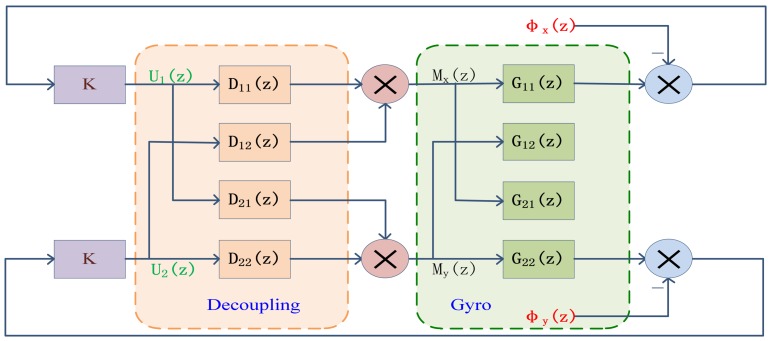
The digital block diagram of the decoupling system.

The digital block diagram of the decoupling system is shown in Figure 13.

The open-loop transfer function matrix of the gyro after decoupling can be expressed as:

(15) $$G_{K}\prime (z)=\left[\begin{array}{cc} G\prime_{K1}(z) & 0\\ 0 & G\prime_{K2}(z) \end{array}\right]=\left[\begin{array}{cc} \frac{KT^{2}(z+1)}{2H{\text{(}z-1)}^{2}} & 0\\ 0 & \frac{KT^{2}(z+1)}{2H{\left(z-1\right)}^{2}} \end{array}\right]$$

By the bilinear transformation, Equation (15) is expressed as:

(16) $$G\prime_{K1}=G\prime_{K2}=\frac{K(1-\frac{T}{2}\omega )}{H\omega^{2}}$$

where $\omega =\frac{2}{T}\frac{z-1}{z+1}$ and T is the sample time.

Similarly, through the correction, we can get the following correction transfer function:

(17) $$G_{J}(\omega )=\frac{16.8(\frac{1}{20}\omega +1)}{\left(\frac{1}{1000}\omega +1)(\frac{1}{15000}\omega +1\right)}$$

Last, *G_J_*(*z*) can be obtained after substituting $\omega =\frac{2}{T}\frac{z-1}{z+1}$ into *G_J_*(*ω*), *i.e*.,:

(18) $$G_{J}(z)=\frac{16.8(33.67z-33)(0.8824z+0.8824)}{\left(z-0.3333)(z+0.7647\right)}$$

**Figure 14.**
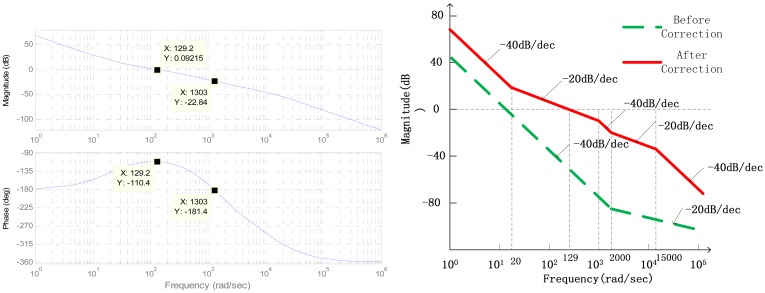
The digital Bode plots of open-loop system.

The open-loop transfer function Bode plots can be seen in Figure 14 after correction.

As seen in Figure 14, the system is not stable before correction because the slope of the amplitude-frequency characteristics crossing 0 dB line is −40 dB/dec, and the slope in high frequency domain is only −20 dB/dec. After correction, it can reach stability because the slope of the amplitude-frequency characteristics crossing 0 dB line is adjusted to −20 dB/dec, and the slope in the high frequency domain becomes −40 dB/s. The crossover frequency can reach 130 rad/s while the phase margin is 70 deg and the magnitude margin is 22.8 dB through the analysis of bode plots in Matlab/Simulink.

### 5.4. Simulation of System

**Figure 15.**
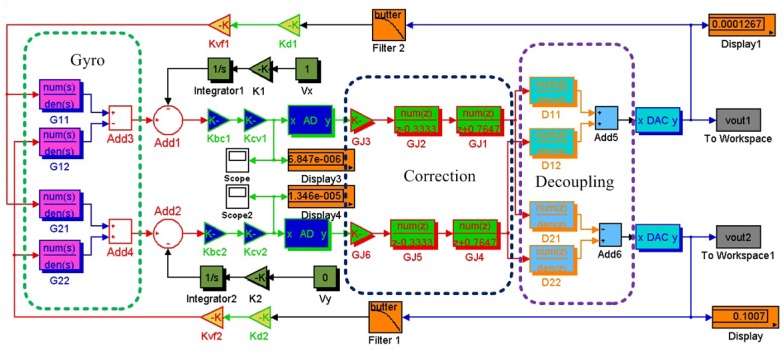
The simulation diagram of closed-loop system.

The simulation diagram of closed-loop system can be seen in Figure 15. The blocks *G_11_*, *G_12_*, *G_21_* and *G_22_* are the gyroscope transfer functions. There are digital correction blocks *G_J_*, including *G_J1_*, *G_J2_*, *G_J3_, G_J4_*, *G_J5_* and *G_J6_*, and digital decoupling blocks *D_11_*, *D_12_*, *D_21_* and *D_22_* between A/D and D/A. The A/D sample time is set 0.001 s with 18 bit quantization resolution, and the cutoff frequency of 4th order Butterworth low-pass filter is 80 Hz. The gyroscope circuit system has two closed loops, *i.e*., one is x-axis closed loop and the other is y-axis closed loop. The parameters of two loops, such as angle to capacitance conversion factor *K_bc_*, capacitance to voltage conversion factor *K_CV_* and voltage to torque conversion factor *K_VF_*, are identical except *D* and *G*, which can be seen in Table 2.

**Figure 16.**
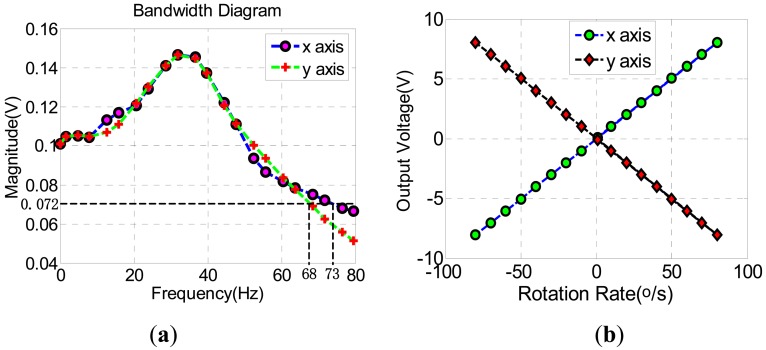
Simulation results diagrams. (**a**) Bandwidths. (**b**) Scale factors.

The bandwiths and scale factors have been simulated in Figure 16, The bandwidth of x-axis closed-loop can reach 73 Hz and y-axis closed-loop is 68 Hz, which can be seen in Figure 16(a). Figure 16(b) shows that the scale factors of x-axis and y-axis closed-loops are 0.1007 V/^o^/s and –0.1007 V/^o^/s, respectively. According the above simulations, the designs of both x-axis and y-axis closed-loop systems are stable enough.

**Figure 17.**
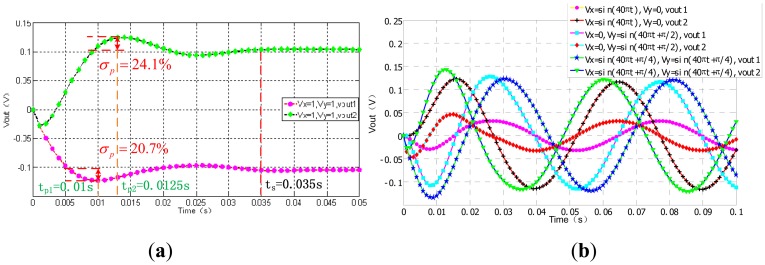
The output of system *versus* the input of Vx and Vy. (**a**) Step response. (**b**) Transient response.

In Figure 17(a), the output curves specfications of the step response can demonstrate the system dynamic performance when Vx = 1 °/s and Vy = 1 °/s. we acquire the overshoots of x and y, are 20.7% and 24.1%, respectively. Both of their adjustment time is 0.035 s. The peak time of vout1 is 0.01 s and the vout2's is 0.0125 s. In Figure 17(b), six transcient response curves with three groups of Vout1 and Vout2 are ploted together for comparison. The sine wave outputs are pure without distortion when Vx = sin(10t)°/s, Vy = 0 and Vx = 0, Vy = sin(10 t +π/2)°/s, and Vx = sin(10 t + π/4)°/s, Vy = sin(10 t + π/2)°/s, respectively. There is a very small amount of cross coupling between x-axis and y-axis closed loops.

## 6. Experimental Results

**Figure 18.**
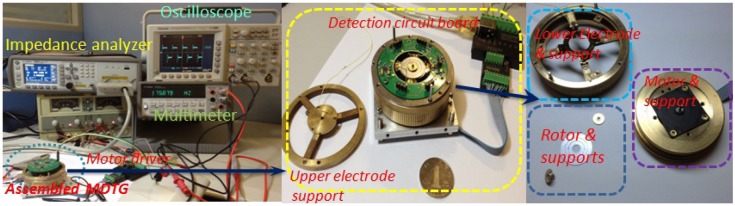
Experimental setup for the adjustable static capacitance of MDTG.

The adjustable effect of the static capacitance has been verified using various pieces of equipment, including an impedance analyzer, an oscilloscope, a multimeter and a power supply shown in Figure 18. A four-layer interface circuit board with the radius of 25 mm is embedded in the assembly. Two diode ring devices HSM2829 (HP Company) are used for demodulation and two AD8221s (ADI Company) are used as the differential signal amplifiers. The complicated leads between upper electrode plate and lower electrode plate are completed through eight pads on both sides of the board. The motor driver can control the speed of miniature motor. The static and dynamic capacitances have been tested via rotating the lower electrode support or replacing the gasket ring with different thickness.

**Figure 19.**
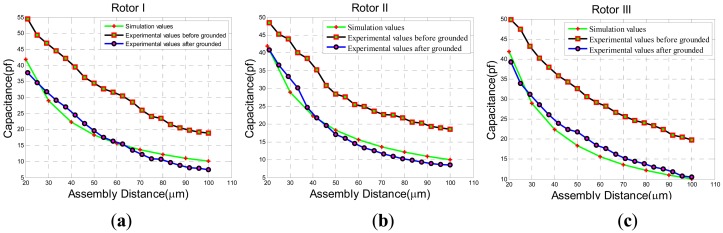
The experimental results of static capacitance. (**a**) Rotor I. (**b**)Rotor II. (**c**) Rotor III.

The experimental results of static capacitance between rotor wafer and the sensing electrode plates can be seen in Figure 19(a) when rotating the lower electrode support one scale of 4.167 μm. Then, the adjustable static capacitance values are compared with the simulation values of sensing electrode plates in Figure 5(b). The curves of capacitance changeable with assembly distance are approximated to the simulation values when the assembly is connected to the ground. Compared with the grounded assembly, there is a constant capacitance value about 10 pF when the assembly is ungrounded. In order to see the possible parasitic capacitance, another two rotor wafers in Figure 19(b) and (c) have been tested. The results are similar to the rotor I. It can be concluded that the assembly has a parasitic capacitance of about 10 pF which is in parallel with the capacitance between the electrode plates and rotor wafer.

**Figure 20.**
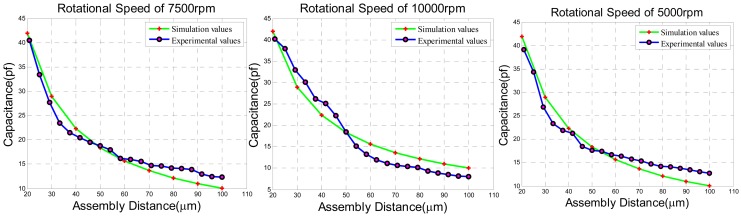
The experimental results of dynamical capacitance under different rotation speed.

The experimental results of adjusting the dynamical capaticance between the rotor wafer and the sensing electrode plates can be seen in Figure 20. The dynamical capacitance values without angular velocity input are compared with the theoretical simulation. The dynamical capacitance has been tested under different rotation speeds of 10,000 rpm, 7,500 rpm and 5,000 rpm when the assembly is connected to the ground to avoid the annoying parasitic capacitance as illustrated above. The results prove that the dynamical capacitance values are approximated to the simulation values regardless of the different rotation speed.

## 7. Conclusions

A novel micro dynamically tuned gyroscope (MDTG) is described from the principle, simulation and fabrication, assembly, circuitry and experimental detail point of view, respectively. Through modal analysis, mechanical and static capacitance simulation, the optimized geometry parameters are derived. Then the fabrication process of rotor wafer, electrode plates and assembly model are investigated step by step. The SEM photos and even the electrical measurement results demonstrate that the proposed design is effective. The static capacitance adjustable assembly model is elaborated to easily tune the static capacitance. First, C/V conversion factors are adjustable because of the changeable static capacitances, which means the scale factors are adjustable conveniently. Second, the optimized gap between electrode plates and rotor wafer can be obtained by experiments. Last, the parallel level between the rotor wafer and electrode plates or the assembly working face can be tested compared with the simulation.

To realize a complete dual-axis gyroscope, a digitalized closed-loop measurement circuit is designed and analyzed. The simulation results can show a satisfactory effect compared with the previous analog design. The discrete correction and decoupling modules are designed to make the closed-loop stable and cross-coupling effect reduced. The system closed-loop bandwidths can reach more than 60 Hz and the dual axis scale factors are completely symmetrical. All the simulation results demonstrate the proposed fabrication of the MDTG can meet the application requirement. In the end, the assembled model is proved effective to achieve the adjustable static capacitance by experiments.

## Figures and Tables

**Table 1.** The simulation parameters.

Name	Outer radius (mm)	Inner radius (mm)	Thickness (μm)
Rotor ring	10	7.0	400
Equilibrium ring	6.9	5.0	400
Inner ring	4.9	2.5	400
Name	Length (mm)	Width (μm)	Thickness (μm)
Torsional spring	1	50	350
Material	Density (kg/m^3^)	Young modulus (Pa)	Poisson's ratio
Si	2,329	1.7 × 10^11^	0.28

**Table 2.** The parameter values of the closed-loop system.

**Parameter**	**Value**
Moment of Inertia (J)	3.2173 × 10^−9^ kg·m^2^
Angular Momentum (H)	6.7512 × 10^−6^ kg·m^2^·rad/s
Sample Time (T)	1/1,000 s
β/C Factor (K_bc_)	6.0478 × 10^−9^ F/rad
C/V Factor (K_CV_)	3.4781 × 10^10^V/F
Power Amplification Factor (K_D_)	10
V/F Factor (K_VF_)	−8.0269 × 10^−8^ N·m/V
Angle to Radian Conversion Factor (K)	π/180
